# Periosteal preservation: a new technique in resection of bone high-grade malignant tumors in children—about eleven cases

**DOI:** 10.1186/s12957-022-02749-1

**Published:** 2022-09-26

**Authors:** Mahmoud Smida, Ameni Ammar, Faten Fedhila, Wiem Douira, Samia Sassi

**Affiliations:** 1 Tunis Faculty of Medicine, Tunis El Manar University, Tunis, Tunisia; 2 Department of Trauma, Orthopedics Kassab Institute, 2010 Manouba, Tunisia; 3 Oncology Unit, Tunis Children Hospital, 1007 Bab Saadoun, Tunis, Tunisia; 4 Department of Radiology, Tunis Children Hospital, 1007 Bab Saadoun, Tunis, Tunisia; 5 Department of Pathology, Salah Azaiez Institute, 1007 Bab Saadoun, Tunis, Tunisia

**Keywords:** Bone sarcoma, Children, Surgery, Limb preservation, Periosteum

## Abstract

**Objective:**

The purpose of this study was to describe a surgical technique of bone resection with periosteal preservation and reconstruction in patients with high-grade bone malignant tumors and to determine its effect on local recurrences, and time and quality of bone union in bone autografting reconstruction.

**Patients and methods:**

We retrospectively reviewed 11 cases of high-grade malignant bone tumors in children aged 4 to 16 years, who were treated with chemotherapy and tumor resection while preserving partially the adjacent periosteum. Tumors were located in the lower limb in eight cases; three tumors were in the humerus. The mean length of the bone defect after resection was 15.8 cm (range, 6–34.5 cm). Reconstruction was provided by non-vascularized autograft in eight cases (lower limb) and polymethyl methacrylate spacer in three cases (upper limb). Patients were followed up for a mean of 71 months.

**Results:**

At the last follow-up, no patients had local recurrence. Three patients were dead because of metastasis. Bone union was good in time and quality in all children who had bone autografting. In cases of PMMA reconstruction, there was periosteal bone formation around the spacer. According to the MSTS functional score, patients with lower limb localizations had a mean score of 27.75 points and patients with upper limb localizations had a score of 24/30.

**Conclusion:**

Preservation of the periosteum in bone resection for malignant tumors could be a good adjuvant alternative for bone reconstruction, without increasing the risk of local recurrence. However, patients must be carefully selected.

## Background

For many years, limb-sparing surgery represents the gold standard for the treatment of patients having primary malignant bone tumors with a survival rate higher than 70% at 5 years [1, 2].

Fear of local recurrence, the bad event, surgeons were prompted at first to perform too large bone resections. In many cases, these excessive and “abusive” resections had removed anatomical structures, which are highly interesting for better anatomical and functional results. The reconstructions were then more difficult with less sure results, a very high rate of complications, and failure. This has prompted surgeons to make resections that are increasingly close to the tumor margins to preserve the maximum amount of normal bone. The periosteum with its capacity for bone regeneration is one of the anatomical structures, which are automatically resected, and currently, no surgeons would think to make resections of high-grade bone tumors while preserving the adjacent periosteum when unaffected.

The purpose of this study was to review a case series of children with high-grade malignant bone tumors managed with a technique of subperiosteal bone resection with preservation of the periosteum and to determine its effect on local recurrences, and time and quality of bone union in patients who have undergone bone autografting reconstruction.

## Patients and methods

Since January 2000, we practiced more than 300 limb preservation surgeries for malignant bone tumors in children. Eleven of these were managed by partially subperiosteal tumor resection by the same surgeon, one of the authors (MS) (Table 1). Following Department Board Staff approval, we retrospectively reviewed this case series.

**Table 1:** Patient characteristics

Patient	Age (years)	Sex	Diagnosis	Location	Side	Particularities	Length (cm) Bone resection	Type SPR	Reconstruction technique and immobilization	Intra-epiphyseal resection
1	14	Boy	Ewing sarcoma	Tibia (proximal)	Left	Skip metastasis	31.5	Type 2	Intercalary prosthesis Cortical autograft (NV) + PC	No
2	8	Boy	Ewing sarcoma	Tibia (distal)	Right	-	15	Type 1	Fibula (NV) Intramedullary nailing + PC	Yes
3	4	Boy	Ewing sarcoma	Tibia (proximal)	Right	Pulmonary metastasis	11.5	Type 1	Cortical autograft (NV) Pin + PC	No
4	8	Boy	Ewing sarcoma	Tibia (proximal)	Left	-	13	Type 1	Cortical autograft (NV) Medialization of the fibula + PC	Yes
5	9	Boy	Ewing sarcoma	Tibia (proximal)	Right	Presence of a second localization (right distal fibula)	6	Type 2	Fibula (NV) Cortical autograft (NV) 2 screws + PC	Yes
6	9	Boy	Ewing sarcoma	Tibia (proximal)	Left	Meta-diaphyseal Distal skip metastasis	10.5	Type 1	Fibula (NV) Cortical autograft (NV) 1 screw + 1 pin + PC	No
7	13	Girl	Ewing sarcoma	Tibia (distal)		-	9	Type 1	Fibula (NV) + PC	No
8	4.5	Girl	Ewing sarcoma	Humerus (distal)	Right	Pulmonary and bone medullary metastasis Pandiaphyseal extension	15.5	Type 2	PMMA spacer 2 nails + TBO	No
9	16	Boy	Osteosarcoma (chondroblastic)	Humerus (proximal)	Right	Pandiaphyseal extension	34.5	Type 2	PMMA Spacer 2 nails + TBO	Yes
10	7	Boy	Metastasis of renal carcinoma	Humerus (distal)	Right	Pandiaphyseal extension	15	Type 2	PMMA Spacer 2 nails + TBO	No
11	13	Boy	Osteosarcoma (fibroblastic)	Femur (distal)	Left	-	12	Type 1	Fibula (NV) LCP plate + PC	No

*NV* non-vascularized, *SPR* subperiosteal resection, *PMMA* polymethyl methacrylate, *PC* plaster cast, *TBO* thoraco-brachial orthosis

Only patients presenting histologically diagnosed high-grade malignant bone tumors of limbs, younger than age 18 years at the time of diagnosis, and who had been managed with partial respect for adjacent periosteum were included in the study. A minimum of 12 months of follow-up was required for patients alive. Benign tumors and recurred malignant tumors were not included in this study.

There were ten boys and two girls with an age ranging from 4 to 16 years at the time of surgery. The tumor was Ewing sarcoma in eight cases, osteosarcoma in two cases, and a bone recurrence of a previously treated clear cell renal cell carcinoma in one case. The tumor was located on the tibia in seven cases, the femur in one case, and the humerus in three cases.

The diagnosis was always made by open biopsies. All patients were managed with pre- and postoperative chemotherapy. In osteosarcoma cases, the two patients received the OS2006 protocol of chemotherapy. They first underwent neoadjuvant chemotherapy based on high-dose methotrexate, ifosfamide, and etoposide. For adjuvant chemotherapy, ifosfamide and etoposide were replaced by cisplatin and doxorubicin in one case because of a poor response to chemotherapy. Patients with Ewing sarcoma were treated with EURO-EWING99 protocol based on vincristine, ifosfamide, doxorubicin, and etoposide (VIDE) as intensive induction chemotherapy and vincristine, actinomycin D, and ifosfamide (VAI) for consolidation therapy. For the case of recurrent clear cell renal cell carcinoma, the patient received the ICE regimen (ifosfamide, carboplatin, and etoposide).

All patients had preoperative magnetic resonance imaging before and after chemotherapy to define the limits of the resection.

### Patients’ selection

Patients were selected for a subperiosteal resection after the study of intra- and extraosseous tumor extension on the MRI exams acquired before and after preoperative chemotherapy. The study encompasses the assessment of the cortical bone and its periosteum. Patients presenting tumors with respect and non-invasion of the adjacent cortex and its periosteum in the two MRI exams were considered potential candidates for subperiosteal resection. The periosteum was considered not involved when a clear normal cortical interface was observed separating it from the tumor; it was considered involved when no normal cortical limits were visualized even on one image.

T1W SE post-Gd enhanced and STIR sequences which display greater contrast between tumoral tissue and cortical bone were useful.

MRI markers for a good response to chemotherapy, assessed on the preoperative post-chemotherapy MRI (a decrease in tumor size and volume), was another encouraging criterion for the selection.

After an explanation and information of the technique’s aims and a description of the involved risks, the patient’s parents gave their consent before their child was operated on.

### Preoperative planning

Before surgery, detailed evaluation was necessary and the MRI images were discussed with the radiologist’s staff. In addition to the longitudinal intraosseous extent of the tumor, and its relationship with the physis, joint, muscle compartments, and neurovascular bundles, we were also concerned with the cortical and periosteal relationships of the tumor.

First, we determined the proximal and distal levels of the bone section in relation to the proximal and distal limits of the tumor.

**Fig. 1:**
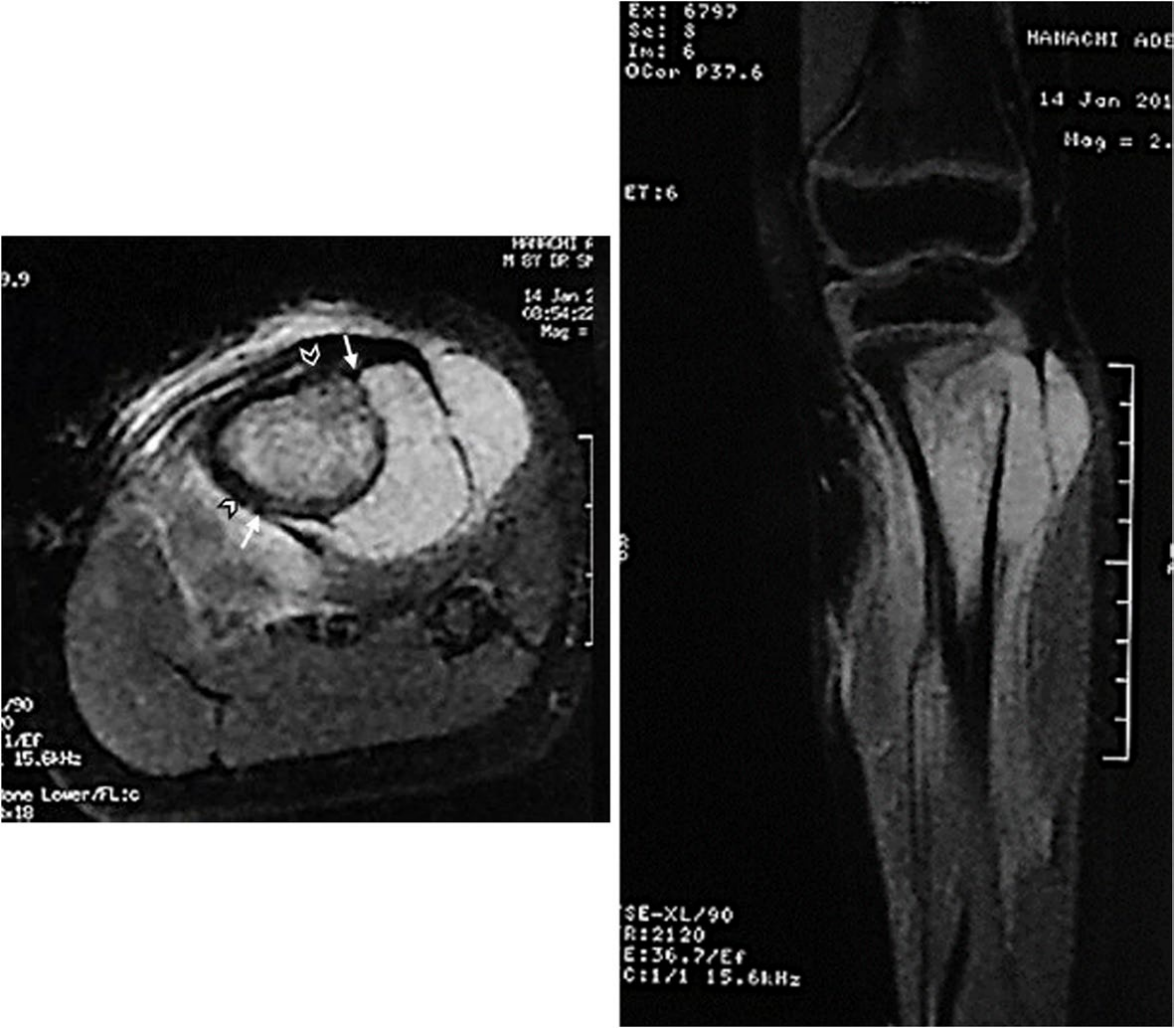
Ewing sarcoma of the proximal tibia in an 8-year-old boy. MR images show an extra-compartmental involvement and a partially circumferential (medial) respect for the CPU. Footnotes: arrows: axial edges; chevrons: level of the periosteal section

Second, we determined the level and direction of the periosteal section. When tumor extension to soft tissues was not circumferential (partially circumferential involvement of the periosteum), we determined on the axial views of preoperative MR images, the two longitudinal lines of the periosteal incisions with 1-cm safe margins from the axial edges of the tumor (Fig. 1). In pure intramedullary tumors with no extension to soft tissues (totally circumferential respect of the periosteum), we determined the horizontal level of the circumferential periosteal section on the affected bone, 1–2 cm from the extraosseous edge of tumor involvement. This surgical planning was correlated with anatomical bony land-markers.

### Surgical technique

Surgery was performed under general anesthesia. A tourniquet was used only for patients with lower limb tumors.

Tumor resection was conventional on the side of the extraosseous involvement and was performed according to the defined principles of previously described surgical techniques [3]. The affected bone was exposed with an excised cuff of normal soft tissue including its periosteum. Concerning the part of the affected bone with only intraosseous involvement and respect for the cortico-periosteal unit, the exposure was continued subperiosteally up and/or down. As planned on MRI images, the periosteum was incised longitudinally at the two axial levels over the bone cortex and then carefully and gently stripped off of the underlying safe cortical bone (type 1 resection). The stripping was performed circumferentially around the safe cortex between the two axial levels along the entire length of the planned resection, leaving in situ, a vascularized periosteal flap.

Two details are very important:

- Periosteal incision and strip exceed the level of the predefined bone section by a few centimeters to avoid injury to the soft tissue by the pneumatic saw.
- The stripped periosteum should not be separated from surrounding soft tissues and muscular attachments.

The osteotomies are planned 1 to 2 cm from the edges of tumor involvement, defined on MRI images.

A technically demanding situation is how to correlate information obtained from the preoperative planning with the real axial tumor limits at the time of surgery. Anatomical landmarks such as the tendons and muscle insertions, tuberosities, bone crests, apophysis, and linea aspera, are used to correlate those previous measurements.

When tumor involvement is only intraosseous with totally circumferential respect for the cortico-periosteal unit, the subperiosteally bone exposure leaves an intact circumferential periosteal sleeve (type 2 resection).

The mean length of the bone defect after resection was 15.8 cm (range, 6–34.5 cm).

After the tumor has been resected, reconstruction of the bone defect was performed using non-vascularized autografts in eight cases. Stabilization was ensured by screws and/or Kirschner wires in six children, an LCP plate in one child, and a custom-made intercalary prosthesis in another child. In the humeral localizations, an intraarticular resection was performed (two distal and one proximal) and a polymethyl methacrylate (PMMA) spacer stabilized with two nails achieved reconstruction. The periosteum has been preserved in order to preserve muscle insertions and to ensure the secondary stability of the PMMA spacer. Primary stability has been entrusted to the nails and orthosis.

In three cases of tibial localization, bone resection was intra-epiphyseal with partial preservation of the growth cartilage in one case. Medialization of the fibula was performed in two cases after tumor resection of the proximal tibia.

The stripped periosteum is then sutured to the muscular aponeurosis or the periosteum in front to obtain a tight tube closure.

For proximal metaphyseal tibial tumors, we reconstruct the extensor mechanism by attachment of the patellar tendon to the periosteum and the aponeurosis of the anterior tibialis muscle.

In the end, sutures were performed on a suction drain. A plaster cast was made to immobilize the operated limb and to maintain easily its alignment. After 8 to 12 weeks, we removed the cast and the patient was allowed full movement but weight-bearing was only permitted after bone consolidation.

For humeral reconstruction, immobilization was done with a thoraco-brachial orthosis for 8 weeks.

The patient with lung metastasis underwent metastasectomy.

### Postoperative evaluation

We have evaluated all patients clinically and radiologically regularly until the final follow-up.

Functional evaluation was done according to the MSTS scoring system [4]. The follow-up radiographs evaluated the consolidation, the extent of bone regeneration, autograft survival and its bone integration, limb alignment and length discrepancy, and local recurrence.

Patients were followed up for a mean of 71 months (range, 12 to 157 months).

Statistical studies were not done because of the small sample size of our casuistry.

## Results (Table 2)

### Clinical assessment

In lower limb localizations, seven children have no difference in the mean range of movement of the treated lower limb compared to the opposite side. The patient with skip metastasis had a moderate limitation of the range of ankle flexions. Six children were even able to participate in some sports activities.

**Table 2:** Results

Patient	Healing time (months)	Follow-up (months)	Complications/bad event	Additional treatment	Limb length discrepancy	Functional score (MTTS)
1	9	157	Breakage of the screws fixing the prosthesis The prosthesis stem had sunk	Complementary autograft Change of the prosthesis by a tibia nail	No	26/30
2	3	153	Proximal graft fracture/pseudarthrosis	Intertibioperoneal grafting Bone lengthening	3 cm	28/30
3	2.5	129	Fracture of the graft	Pulmonary metastasectomy	No	26/30
4	3	131	Fracture of the graft	Bone lengthening	3 cm	28/30
5	2	49	Brain metastasis 42 months after tumor resection Death 9 months after metastasectomy	Metastasectomy	2 cm	28/30
6	2.5	25	-	-	No	30/30
7	4	15	-	Radiotherapy (postoperative)	No	28/30
8	3	12	Pulmonary and multiple bone metastasis Death	-	2 cm	26/30
9	3	33	Migration of the nails from the residual distal epiphysis	Replacement of nails was proposed but refused by the patient	No	23/30
10	3	41	Pulmonary metastasis Death	-	3cm	23/30
11	3	37	-	-	4.5cm	28/30

Children who had humerus tumors presented a limitation respectively in the elbow and shoulder function.

No patient had pain or joint instability in the involved limb.

According to the functional score of the Musculoskeletal Tumor Society, patients with lower limb localization had a mean functional score of 27.75 points (range, 26 to 30 points) and patients with upper limb localizations had a score of 24/30.

### Radiographic assessment

**Fig. 2:**
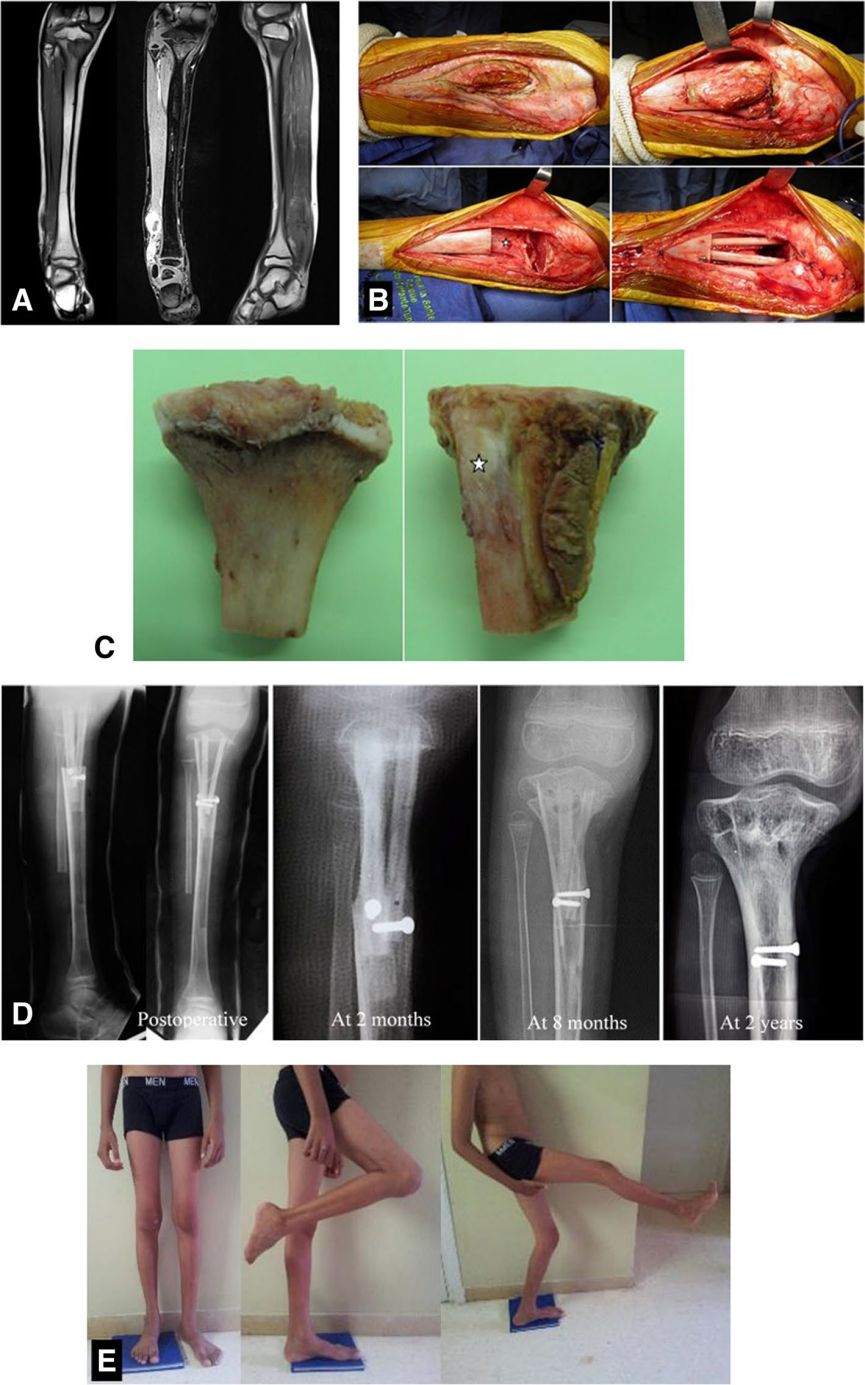
**A** Ewing sarcoma of the distal right fibula with purely intraosseous second localization in the homolateral proximal tibia in a 9-year-old boy. **B** Subperiosteal resection of the tibial localization with the previous site of biopsy. Tripod reconstruction was performed with non-vascularized autografts (2 fibulas and one tibia cortical). The distal fibula tumor was removed (at the same surgical time) (footnote: star: periosteum preserved). **C** Gross specimen with posterior and medial aspects. Proximal tibia without its periosteum and only the previous site of biopsy was resected with the tumor (footnote: star: anterior tibial tuberosity). **D** Serial radiographs showing rapid bone consolidation and good reconstruction. **E** Good functional result at 3-year follow-up. Little lower limb discrepancy. MSST=28/30

**Fig. 3:**
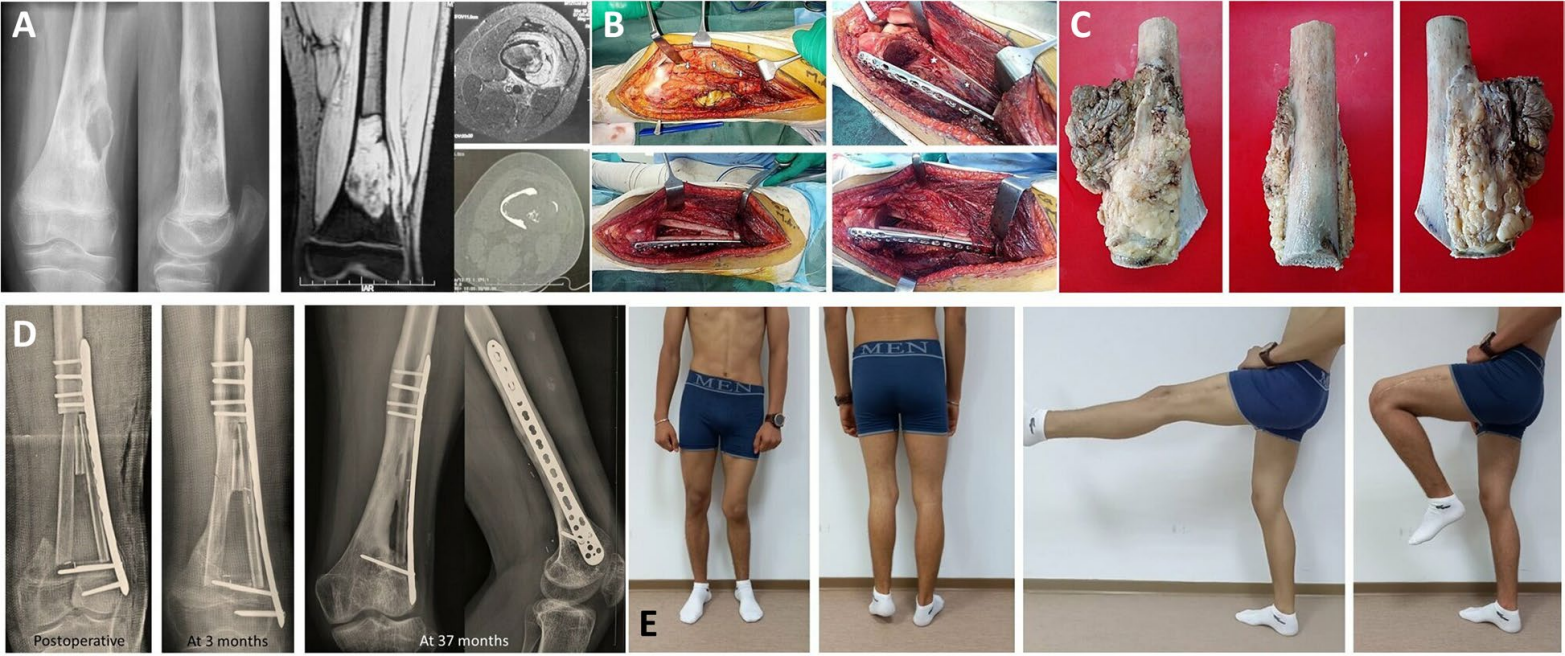
**A** Osteosarcoma of the distal left femur in a 13-year-old boy. **B** Tumor resection by lateral approach with medial periosteal preservation. Reconstruction was performed with two non-vascularized fibula autografts. Only the medial fibula autograft was recovered by the preserved periosteum (footnote: arrows: section line of the periosteum, stars: periosteum preserved). **C** Gross specimen with medial half of femur without its periosteum. **D** Serial radiographs showing rapid bone consolidation and good reconstruction, particularly of the medial aspect. We note the difference between the anatomical results of the two non-vascularized fibula autografts: better union and bone integration of the medial fibula which was recovered by the preserved periosteum. **E** Good functional result at 3-year follow-up with 4.5-cm limb discrepancy and left genu valgum. MSST=28/30

We observed a periosteal new regenerated bone on plain radiographs at mean 3 months after surgery. It gradually united with the grafted bone that remodeled to a normal bone having a cortex and medullary canal (Figs. 2 and 3). The regenerated bone appeared irregular in two patients and was related to the peroperative periosteum tear after metaphyseal tumor resection.

All the patients’ grafts had completely healed, with a mean time of 3.45 months (range, 2–9 months). In the patient with a closed growth plate and the largest intercalary bone resection length (31.5cm), bone union was weak at 9 months and a complementary autograft was necessary. However, in those with an open growth plate, this occurred at a mean of 2.6 months (range, 2–3 months).

**Fig. 4:**
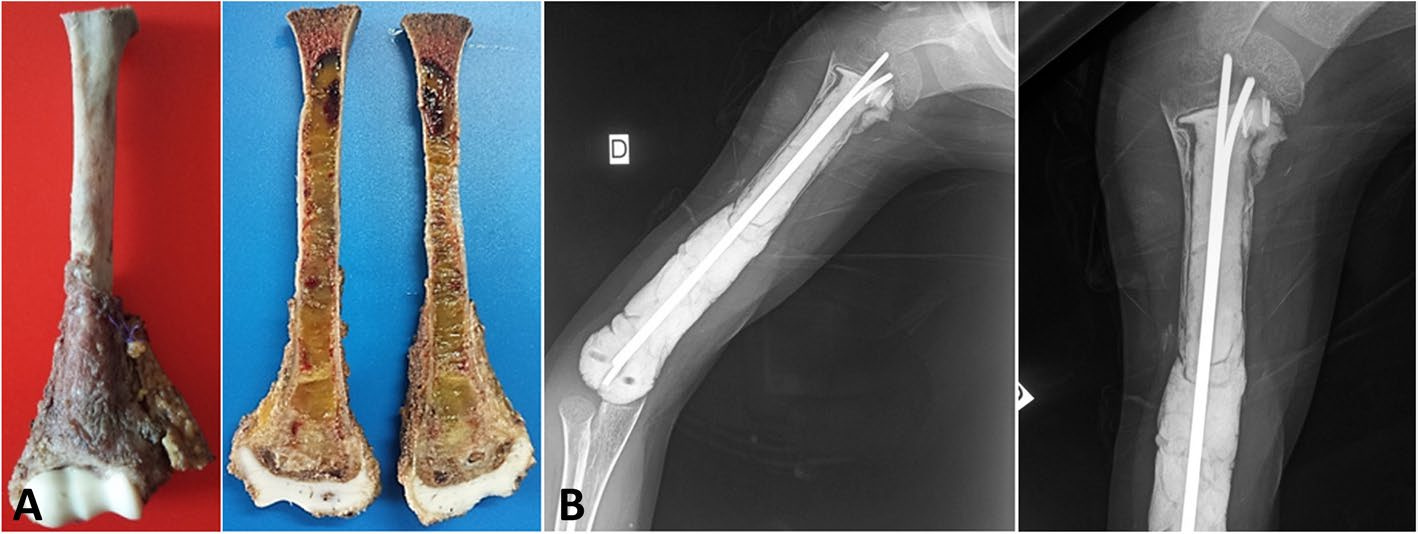
**A** Pandiaphyseal extension of bone recurrence of a previously treated clear cell renal tumor. Type 2 subperiosteal resection. **B** Periosteal bone reconstruction around the PMMA permitting its good fixation

In the cases of humerus localizations, the periosteal bone reconstruction around the PMMA was visible at 3 months (Fig. 4). It was good in two cases (patients 8 and 10) and poor in the other (patient 9).

### Oncologic results

The resection histological margins were negative for tumors in all patients particularly on the side of the subperiosteal resection.

According to Huvos et al. criteria [5], response to chemotherapy was good in nine patients and poor in two cases (one osteosarcoma and one Ewing sarcoma). The patient with Ewing sarcoma had received a dose of 45 Gy postoperative radiotherapy.

At the final follow-up evaluation, there was no local recurrence in all patients and eight patients continued to be disease-free. However, three patients died of distant metastases with no local recurrence.

### Complications

No infection or nerve palsy had occurred in all patients.

A fracture of the graft occurred in three patients resulting from early activity and resolved by immobilization in two cases. In the third case, the fracture was complicated by a pseudarthrosis that required bone grafting.

The patient with skip metastasis required the addition of a cancellous bone graft and replacement of the prosthesis by a tibia nail.

Limb length discrepancy was noted in six patients. Four patients had lower limb discrepancy with a shortening of the operated limb (2–4.5 cm) including the three patients who had trans-epiphyseal resection. Bone lengthening was successfully done for two patients.

No complication occurred at the donor sites.

## Discussion

Limb-salvage surgery has become a standard of care for the treatment of most primary malignant bone tumors. A safe-margin resection of the tumor represents the first goal of this surgery. Bone reconstruction and consolidation that retain a functional limb represent the second goal, which becomes a therapeutic challenge particularly when bone defects are large after tumor resection.

Optimal and successful surgical removal of a primary malignant bone tumor requires two, partially conflicting, prerequisites:

- A recommended large resection with a thick surrounding rim of normal soft tissue to avoid local recurrence
- An increasingly adopted economic resection with the removal of cortex and periosteum, to allow a better function [6]

How to find the right balance is a main question and a big challenge for the onco-surgeon when planning the surgical treatment.

Common bone reconstruction options include numerous surgical techniques: autogenous grafting (vascularized or not), allografts, induced membrane technique, endoprosthesis, bone transport (Ilizarov technique), and extracorporeal devitalized resected tumor including irradiation, autoclaving, pasteurization, low-heat, or freezing with liquid nitrogen. In young patients with the potential for long-term survival, biologic reconstructions are often recommended [7, 8]. However, because of limited success and/or complications, none of these methods has proven to be the ideal choice [9].

Diminished biological ability is the major inconveniences of non-vascularized grafts [10, 11]. Reconstruction with allografts has many problems such as fracture, infection, and nonunion and continues to be challenging [12, 13]. The use of vascularized autograft alone or in combination with an allograft is difficult and requires considerable technical expertise that is not always available. Moreover, these techniques need prolonged immobilization for bone consolidation [14]. Induced membrane technique is associated with an elevated nonunion rate and graft resorption [15, 16]. The Ilizarov technique is not preferred by many surgeons in reconstructions after the resection of bone tumors. Moreover, it has many complications including delayed ossification and maturation, nonunion, and bone resorption [17, 18]. Because of its complications particularly aseptic loosening and infection, prosthetic reconstruction had a high failure rate at 5 to 10 years of follow-up [19, 20]. Extracorporeal devitalized autografts have the same complications as allografts but their major disadvantage is related to the absence of material for the histological examination of the effect of chemotherapy and the determination of surgical margins.

The extent of bone and soft tissue removed during tumor resection is a major cause of high rates of failure and complications [21, 22]. Currently, to decrease the risk of these complications, surgeons attempt to preserve host tissues during tumor resection by reducing the margins of resection [23, 24]. The optimal margin for a given tumor is yet unknown. A few years ago, surgeons have performed wide resections with a large margin of safety (5 cm), thus removing a lot of normal bones and soft tissues. With the appearance of numerous complications, the requirement for broad surgical margins has decreased to 2–3 cm [25] and then to 1–2 cm [26, 27]. Encouraged by the oncological results, surgeons have developed new audacious techniques like multiplanar osteotomies and hemicortical resections with limited margins [23, 28] and epiphyseal preservation in the surgery of metaphyseal malignant bone tumors [27, 29]. With the absence of local tumor recurrence and the advent of robotic-assisted surgery and navigated osteotomies, bone resection margins are measured now in millimeters [28, 30, 31].

Ideal bone reconstruction would drastically decrease the incidence of such aforementioned complications. It needs a number of requirements: biological affinity, resistance to infection, sufficient biological strength, and durability [24]. Preservation of the periosteum responds very well to these requirements and it is well known to be ideal in regenerating a new living bone with sufficient strength and durability [32].

The osteogenic potential of the periosteum has long been recognized [33]. Animal experimentations have demonstrated that it can restore an entire bone defect, whether parcel or segmental, regardless of the length removed [34, 35]. This good biological characteristic has given rise to some applications in human therapeutics [32, 36]. Furthermore, the healing of large defects by an intact periosteal sleeve has been reported in bone fractures in children [9, 37].

The periosteum surrounding bone is an important biological structure having properties that change with age. In children, the periosteum is thick and has two distinct layers. The inner cambium layer contributes to the new bone formation by providing both of the critical substrates for osteogenesis: a population of osteoprogenitor cells and a rich plexus of blood vessels. This osteogenic ability of the periosteum decreases with age. The fibrous layer, comprised mainly of collagen, provides strength and can provide some stability in the treatment of pediatric fractures and bone reconstruction [34, 37].

Several authors have practiced the technique of subperiosteal resection for solitary and aneurysmal bone cysts [38–41] and also for benign bone tumors [14, 42, 43] as a sure prophylactic method against local tumor recurrence.

There are a number of advantages of periosteal preservation when resecting benign bone tumors in children. Their periosteum is a thick, strong membrane that strips easily and is also easily sutured. Preservation of the integrity of the periosteum after subperiosteal resection constitutes a valuable matrix for bone regeneration. It rapidly produces new bone, which successfully stabilizes the defect in a short time [38]. Keeping the periosteal sleeve allowed the osteoprogenitor cells from the inner layer to invade the hematoma in the tightly sutured periosteal tube [39].

Furthermore, preservation of muscle attachment to the periosteum after subperiosteal resection could improve the short- and long-term functional outcomes and maintain the dynamic stability of the adjacent joints [39]. Subperiosteal resection was proposed by Shoji et al. [40] for large aneurysmal bone cysts of the distal fibula in an attempt to preserve attachments of the lateral ligaments to the periosteal sleeve maintaining lateral ankle stability.

Several surgeons bridge the bone defect with a bone graft or bone substitute to keep the periosteal tube expanded preventing thereby the production of an hourglass constriction and avoiding the collapse of the soft tissue into the bone defect [44]. In these cases, preservation of the periosteum constitutes an important factor for consistent incorporation of the graft and excellent remodeling [43]. In his study, Mostafa [39] did not apply any bone graft, and to obtain a uniform bone reconstruction and avoid irregular healing, he sutured the redundant periosteum that remained after the excision of large cysts over a rolled gel foam.

Periosteal preservation is advantageous in terms of consolidation of bone reconstruction. Healing of the bone defect occurs in a progressive manner and osteogenesis occurs initially at the margins of the cavity and moves toward its center. Progressive calcification follows then giving rise to a solid bone.

Despite the deleterious effects of chemotherapy agents on bone healing, the healing time in our patients was 3.45 months (range, 2–9 months). It was comparable to that of subperiosteal resection of benign bone cysts and tumors, varying from 4 to 6 months [38, 39, 43], and to that of the induced membrane technique ranging from 4 to 8 months [8, 45].

Appreciating the role of the periosteal sleeve in the reconstruction of bone defects, and because of the histological and immuno-chemical similarities with the periosteum, several authors favor the induced membrane among the biological reconstruction techniques [8, 45]. Pelissier et al. [46] showed that these membranes have a rich capillary network and high concentrations of growth and osteoinductive factors with osteogenesis-improving capabilities.

Based on the current surgical trend of minimal safe surgical margins and the aforementioned complications associated with other techniques of bone reconstruction and advantages of periosteal preservation, this new surgical procedure of subperiosteal resection of malignant bone tumors was developed exploiting the intrinsic osteogenic potential of the periosteum to bridge bone defects.

However, inadequate and marginal tumor resection is associated with a high risk of local recurrence that carries a dismal overall prognosis [1, 2].

The extent of successful surgical technique in limb-sparing surgery is always related to the local recurrence rate. Resection of a bone tumor with its adjacent periosteum is widely practiced and recognized safe procedure with an acceptable local recurrence rate. Our technique avoids the loss of the periosteum and no previous study has shown the possibility of periosteal preservation in the resection of malignant bone tumors.

Anatomic compartments have natural temporary barriers to occult tumor spread, and in bone, the cortex has a tendency to act as one of these barriers [3]. We believe that when the cortex is not crossed by the sarcoma, subperiosteal resection can be able to achieve local control of bone sarcoma and may provide a safe margin of resection to prevent tumor reoccurrence; the extra-periosteal margin suggested by all authors may be then unnecessary. This view is supported by the fact that no tumor recurred locally in the retained periosteum at the last follow-up in all children in our study.

To avoid local recurrence, a disastrous event for the patient and the medical staff, it is necessary to select carefully patients for subperiosteal resection of malignant bone tumors. Meticulous analysis of the tumor and its adjacent cortex and periosteum of the MRI images is then necessary. With its high level of soft tissue resolution, it allows a clear delineation of cortical and soft tissue extension [6, 47–50] and may elucidate transcortical infiltration and periosteal extension [51]. Bloem et al. [48] have prospectively evaluated the relative value of MRI and computed tomography (CT), in local tumor staging in 56 patients with primary bone sarcoma. The results of imaging were correlated with findings at the histological study of the resected specimens. The authors have found that MRI was significantly superior to CT in defining intraosseous tumor length and was as accurate as CT in demonstrating cortical bone involvement. The involvement of cortical bone was identified on MRI by the replacement of the signal void of the cortical bone by the increased signal intensity of the invading tumor. Identification of cortical tumor crossing should include areas of signal intensity that may turn out to be edema upon pathologic evaluation of the specimen. In the study of Golfieri et al. [52], the STIR sequence demonstrated an ill-defined increased SI in surrounding muscles that subsequently proved not to be infiltrated by the tumor in 37 of 54 cases of malignant bone tumors. The change on the STIR sequence was attributed to tissue edema and could be differentiated from direct tumor invasion by the absence of mass effect and by a slightly reduced SI compared with the tumor. Moreover, the STIR sequence appeared to be more sensitive than the short TR/TE SE scan to the spread of tumor beneath the periosteum, into adjacent soft tissues.

The technique outlined is relatively simple for an experienced surgeon but it requires careful consideration and stringent preoperative criteria for selecting candidate patients.

Generally, there are three types of relationships between tumor and periosteum according to the MRI aspect of the cortico-periosteal unit (CPU):

1. Type 1: cases in which the CPU (cortex and thereby periosteum) is not in contact with the tumor (for example, skip lesions or metaphyseal eccentric tumors without endosteal contact with the opposite cortex)
2. Type 2: cases in which the tumor has an endosteal contact but without cortical crossing and periosteal invasion (for example, purely intra-compartmental tumors: stages IA, IIA, and IIIA of Enneking surgical staging system [3] or height diaphyseal invasion of metaphyseal tumors)
3. Type 3: cases in which the CPU is crossed entirely by the tumor that invades the periosteum

Subperiosteal resection may be safely adopted in type 1. For type 2, if cortico-periosteal invasion is doubtful on MRI, particularly after chemotherapy, the possibility of any tumoral cell passing the cortex is great and then periosteal preservation is contraindicated. However, if the periosteum is unscathed on the MRI, we can try to preserve it. In type 3, subperiosteal resection is not possible and is contraindicated.

For precise surgical guidelines, further histological studies will have to be carried out in comparison with the MRI data.

The main limitations of our study are the low patient number and its retrospective character. Although we obtained a high reconstruction potential of this operative technique, further studies are necessary to confirm our results. Furthermore, further follow-up assessment is required to determine the long-term rates of local recurrence.

In conclusion, periosteal preservation surgery is an effective limb-saving technique to treat high-grade malignant bone tumors in children, without increasing the risk of local recurrence at short and medium terms. No previous study has highlighted the effectiveness of the periosteum in bone reconstruction and consolidation after resection of malignant bone tumors. An intact periosteum in connection with its peripheral tissues offers the benefit of maintaining a potentially biologic reconstruction oriented to the present demand in orthopedic oncology, i.e., a good, fast, and permanent reconstruction for better function in children who have the potential for long-term survival. The technique is proved to be safe and not complicated and avoids morbidities associated with other techniques. However, patients should be carefully selected and the procedure should be considered only when strict indications are satisfied.

## Data Availability

The datasets used and/or analyzed during the current study are available from the corresponding author on reasonable request.
