# Correction: Periosteal preservation: a new technique in resection of bone high-grade malignant tumors in children—about eleven cases

**DOI:** 10.1186/s12957-023-03040-7

**Published:** 2023-05-24

**Authors:** Mahmoud Smida, Ameni Ammar, Faten Fedhila, Wiem Douira, Samia Sassi

**Affiliations:** 1 Tunis Faculty of Medicine, Tunis El Manar University, Tunis, Tunisia; 2 Department of Trauma, Orthopedics Kassab Institute, 2010 Manouba, Tunisia; 3 Oncology Unit, Tunis Children Hospital, Bab Saadoun, 1007 Tunis, Tunisia; 4 Department of Radiology, Tunis Children Hospital, Bab Saadoun, 1007 Tunis, Tunisia; 5 Department of Pathology, Salah Azaiez Institute, Bab Saadoun, 1007 Tunis, Tunisia

**Correction: World J Surg Onc 20, 312 (2022)**

**https://doi.org/10.1186/s12957-022-02749-1**

Following publication of the original article [1], the author reported that the arrows, chevron and stars in Figs. 1 and 2 were removed during the corrections stage. These were added back to the images and the original article has been updated.

**Fig. 1:**
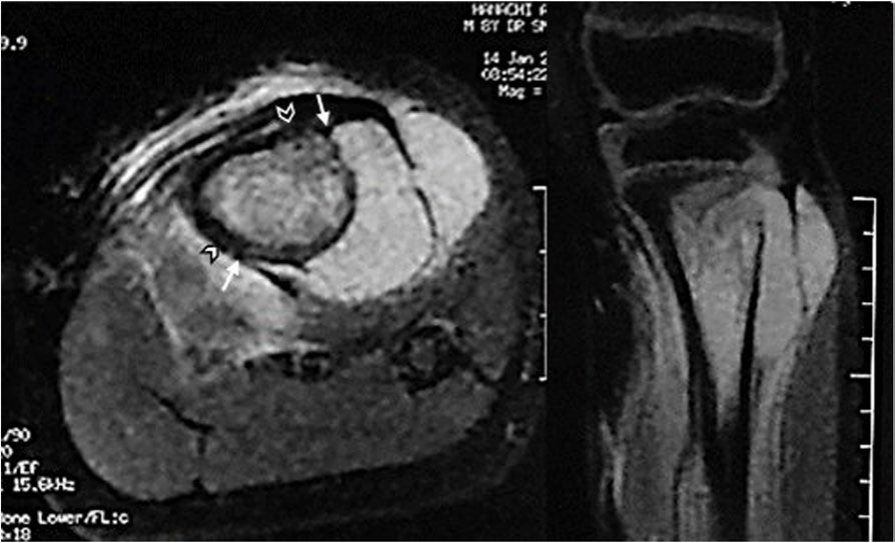
Ewing sarcoma of the proximal tibia in an 8-year-old boy. MR images show an extra-compartmental involvement and a partially circumferential (medial) respect for the CPU. Footnotes: arrows: axial edges; chevrons: level of the periosteal section

**Fig. 2:**
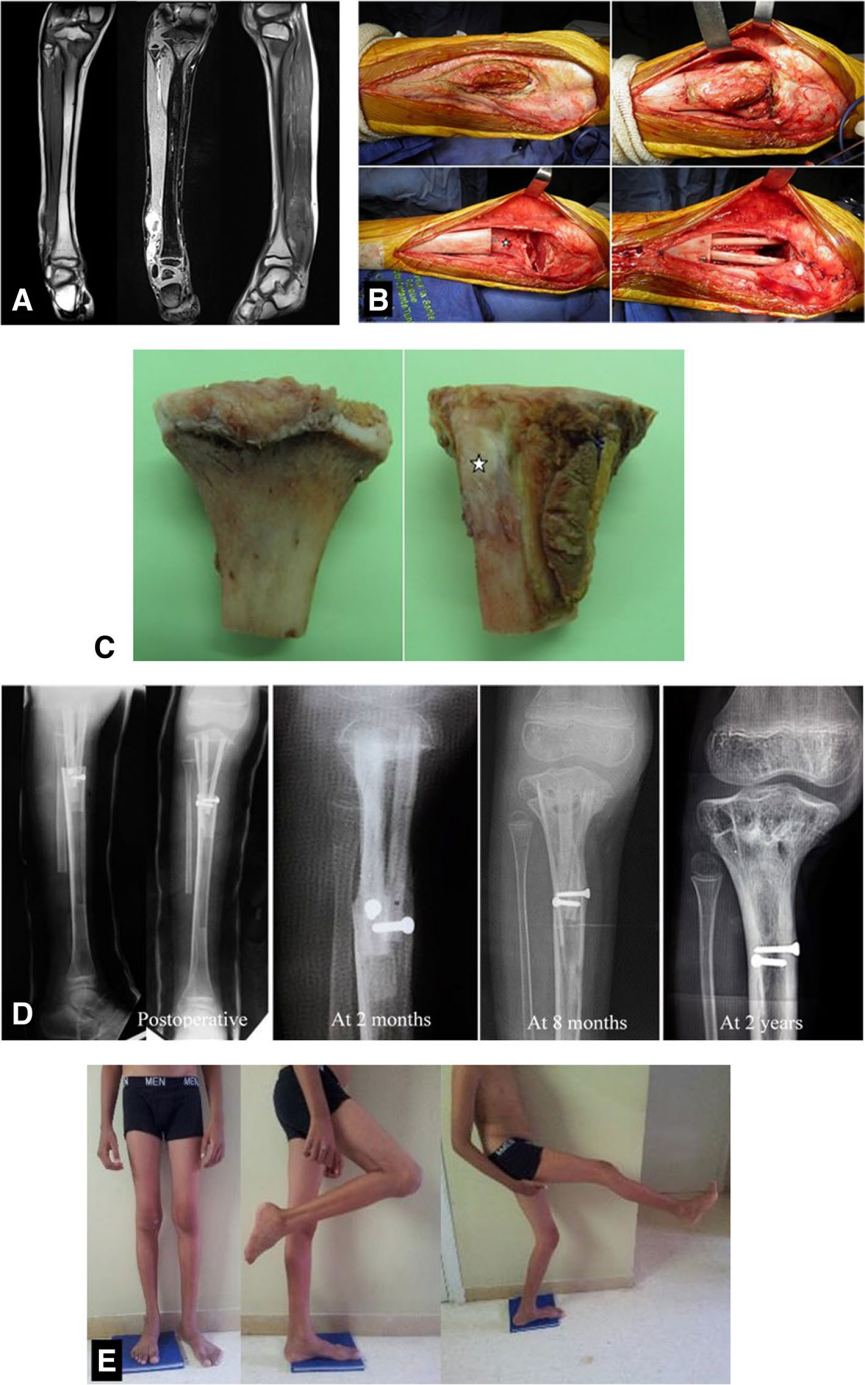
**A** Ewing sarcoma of the distal right fibula with purely intraosseous second localization in the homolateral proximal tibia in a 9-year-old boy. **B** Subperiosteal resection of the tibial localization with the previous site of biopsy. Tripod reconstruction was performed with non-vascularized autografts (2 fibulas and one tibia cortical). The distal fibula tumor was removed (at the same surgical time) (footnote: star: periosteum preserved). **C** Gross specimen with posterior and medial aspects. Proximal tibia without its periosteum and only the previous site of biopsy was resected with the tumor (footnote: star: anterior tibial tuberosity). **D** Serial radiographs showing rapid bone consolidation and good reconstruction. **E** Good functional result at 3-year follow-up. Little lower limb discrepancy. MSST = 28/30
